# The Use of Perpendicular Reconstruction Plates for the Buttressing of the Quadrilateral Surface Fractures of the Acetabulum: An Easy and Low-Cost Choice

**DOI:** 10.7759/cureus.73957

**Published:** 2024-11-18

**Authors:** Ioannis P Galanopoulos, Georgios-Iason Oikonomou, Panagiotis Drakopoulos, Spyridon A Psarakis

**Affiliations:** 1 Orthopaedic Department, Thriasio General Hospital, Athens, GRC; 2 Laboratory for the Research of the Musculoskeletal System, University of Athens, KAT Hospital (Attica General Hospital), Athens, GRC

**Keywords:** acetabular fracures, low-cost technique, pelvic acetabular trauma, quadrilateral surface, reconstruction plates

## Abstract

The quadrilateral surface fractures of the acetabulum are becoming more frequent due to the aging population. The optimal fixation of the quadrilateral surface through an appropriate surgical approach and a reliable and effective technique presents a challenge for orthopedic trauma surgeons. In this study, we present the results of 12 patients treated in our department who underwent internal fixation of the quadrilateral surface with two reconstruction plates in a vertical orientation for adequate fracture buttressing. The clinical and radiological outcomes are encouraging, and we believe that this low-cost technique can be a reliable method for optimal fixation of acetabular quadrilateral surface fractures.

## Introduction

Fractures of the quadrilateral surface of the acetabulum are becoming increasingly common, especially among the elderly, who represent a growing subgroup of pelvic trauma patients. This has led to a demand for effective treatments and the development of novel surgical techniques and implants [1]. While the anterior intrapelvic approach is well-established, its benefits and limitations are still evolving. Additionally, various fixation methods and implants have been introduced [2]. This study presents the outcomes and clinical experience of treating quadrilateral surface fractures with two reconstruction plates arranged perpendicularly.

## Materials and methods

This is a retrospective analysis of 12 patients (10 males, two females) diagnosed with acetabular quadrilateral surface fractures, all of whom underwent surgical intervention at our institution between January 2022 and October 2024. The patients had a mean age of 48 years (range: 18-84 years). Nine patients sustained high-energy trauma, while three experienced low-energy injuries secondary to osteoporosis. All surgical procedures were performed by a single, experienced surgical team using a modified Stoppa approach. In cases involving complex fractures, additional approaches such as the lateral window of the ilioinguinal or Kocher-Langenbeck approach were employed. These included fractures that were not limited to isolated quadrilateral surface fractures but also involved multifocal acetabular injuries.

Surgical technique

**Figure 1:**
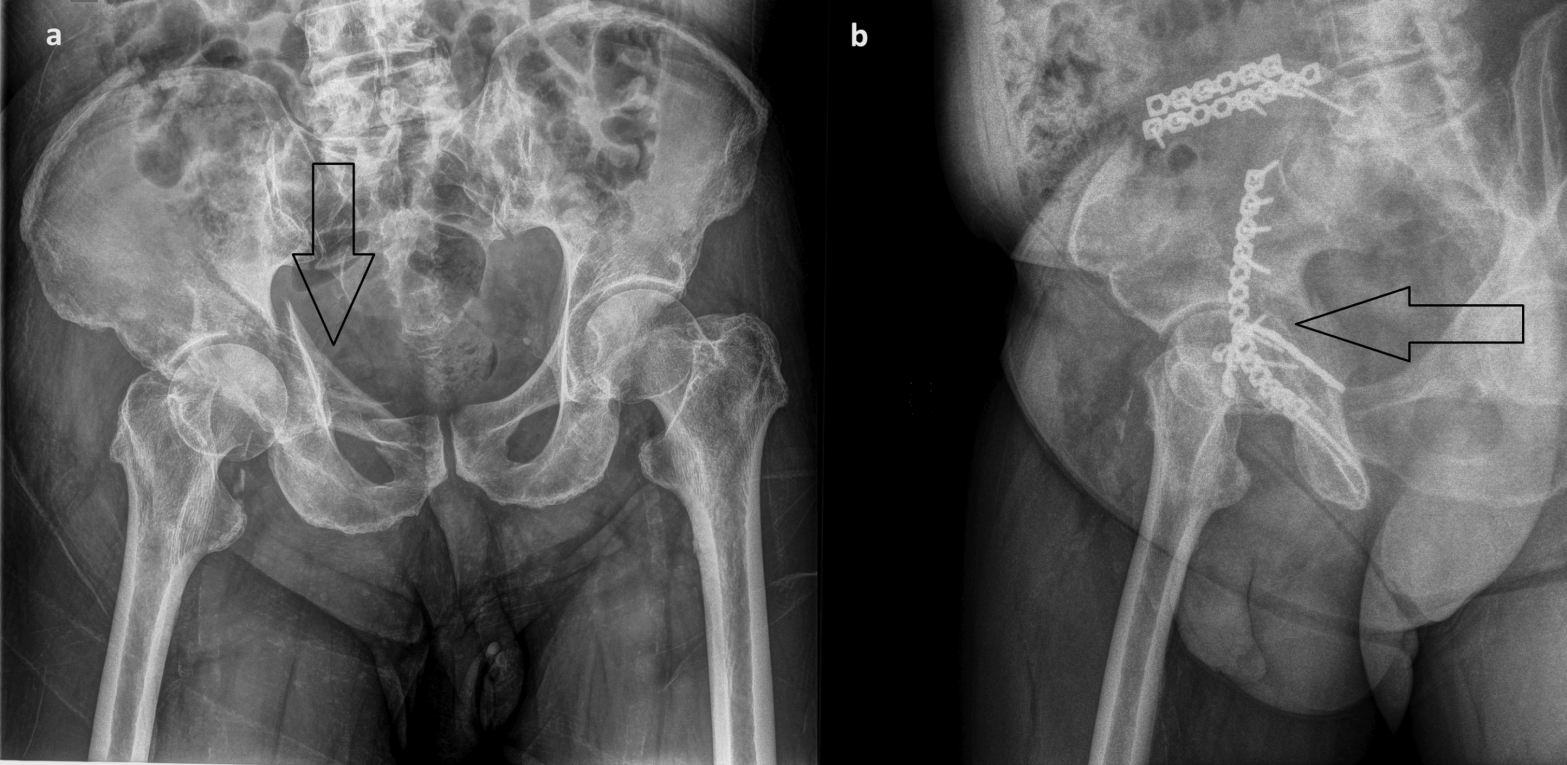
Fracture of the quadrilateral surface of the acetabulum: (a,b) fixation with two perpendicular reconstruction plates.

**Figure 2:**
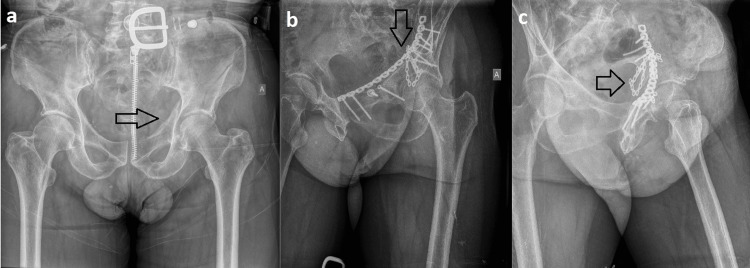
Fixation of the quadrilateral surface with a suprapectineal reconstruction plate and additional calcaneal plate for adequate buttressing: (a) preoperative image; (b) postoperative image - obturator oblique view; (c) postoperative image - iliac oblique view

For fracture fixation, we utilized a dual 3.5 mm reconstruction plate construct: a precontoured suprapectineal plate combined with an intraoperatively contoured plate placed vertically in a T-shape configuration for buttressing the quadrilateral surface (Figure 1). In two patients, alternative constructs were used. One patient received a calcaneal plate combined with a suprapectineal reconstruction plate, while the second patient had two longitudinal plates (suprapectineal and infrapectineal) placed in an anterior-posterior orientation (Figure 2).

**Figure 3:**
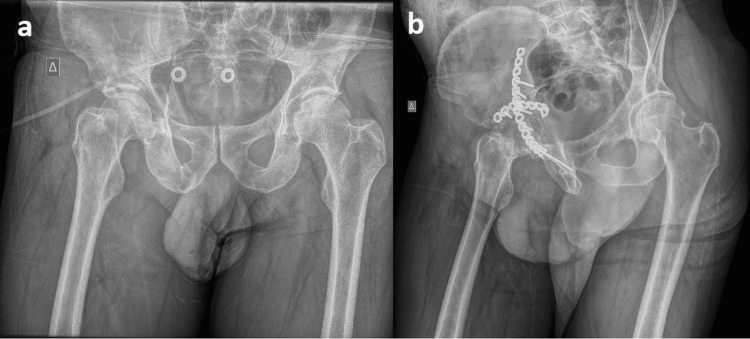
Perpendicularly placed reconstruction plates for the quadrilateral surface buttressing: (a) preoperative image; (b) postoperative image

This technique does not require specialized or high-cost implants, demonstrating the effectiveness of readily available, cost-efficient materials for stabilizing quadrilateral surface fractures (Figure 3).

Postoperative care

All patients followed a standardized rehabilitation protocol, which included non-weight-bearing mobilization for the first 12 weeks post-surgery, with progression to partial weight-bearing as tolerated thereafter. Patients were monitored with monthly radiographic follow-up for the first six months to assess fracture healing and implant positioning. A comprehensive clinical assessment, including radiographs and Harris Hip Score (HHS) evaluations, was performed at one year postoperatively.

## Results

All patients treated with two perpendicular reconstruction plates exhibited favorable outcomes, with adequate support for the quadrilateral surface (QLS). None of these patients developed post-traumatic arthritis or femoral head protrusion. Additionally, the patient who received a calcaneal plate combined with the reconstruction plate for QLS buttressing demonstrated similarly positive outcomes.

**Figure 4:**
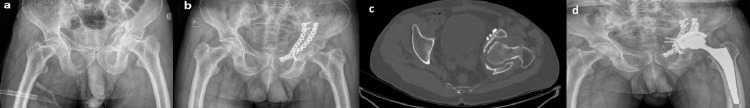
Insufficient buttressing of the quadrilateral surface and total hip arthroplasty as final solution: (a) preoperative image; (b) early postoperative image with good reduction; (c) CT image eight months postoperatively shows inadequate buttressing of the quadrilateral surface and femoral head protrusion; (d) total hip replacement as final solution

In contrast, the patient treated with two longitudinal plates - one suprapectineal and one infrapectineal, positioned from anterior to posterior - experienced several complications. These included femoral head protrusion, leg length discrepancy, and limping caused by insufficient abductor function, which led to disturbances in hip biomechanics. Due to these complications, this patient ultimately required a total hip arthroplasty (Figure 4).

## Discussion

The proper fixation of the quadrilateral surface (QLS) in acetabular fractures is critical, particularly as the incidence of such fractures increases among older adults. Recent studies have addressed the complexity of managing these fractures, emphasizing the need for a reliable classification system and effective surgical approaches.

A notable contribution to the management of quadrilateral surface fractures comes from Kaifang Chen et al. who proposed a new classification for QLS fractures after identifying limitations in the established Judet-Letournel system. By analyzing data from over 1,000 patients across eight trauma centers, the study concluded that their new classification system provided greater reproducibility and reliability, facilitating more accurate fracture assessment and guiding surgical intervention [3]. Surgical strategies varied depending on fracture patterns, with a preference for the anterior intra-pelvic approach (modified Stoppa technique) for QLS fixation, particularly when medial buttressing was necessary.

Various fixation techniques have evolved over the past few decades, with advancements in technology allowing for the development of specialized implants and improved surgical outcomes. For instance, the introduction of the anterior intrapelvic approach by Cole and Bolhofner in 1994 provided a less invasive alternative to the traditional ilioinguinal approach, allowing direct access to the quadrilateral surface while minimizing soft tissue disruption [4].

Other studies have also emphasized the importance of adequate buttressing for the quadrilateral surface. Schäffler et al. highlighted the need for medial support in cases of femoral head displacement, demonstrating that conservative management or insufficient fixation often led to poor outcomes, including the need for secondary reconstructive surgeries [5]. These findings support the importance of proper QLS stabilization, as insufficient fixation can result in femoral head protrusion, leg length discrepancy, and gait disturbances, as seen in our own series. Notably, one patient in our cohort required total hip arthroplasty due to complications related to inadequate QLS fixation.

Our study supports the effectiveness of the dual reconstruction plate technique, which has been widely used in the management of acetabular fractures involving the QLS. We observed favorable outcomes in patients treated with two perpendicular reconstruction plates, with no instances of post-traumatic arthritis or femoral head protrusion. This finding is consistent with the results of Ciolli et al., who reported successful outcomes using the suprapectineal quadrilateral surface plate via the anterior intrapelvic approach. They found that this fixation technique effectively prevented medial subluxation of the femoral head, particularly in elderly patients, and was associated with good clinical and radiological outcomes [6].

In contrast, complications associated with the use of two longitudinal plates in one of our patients underline the potential risks of inadequate or mismatched fixation. This patient developed femoral head protrusion and leg length discrepancy, ultimately requiring a total hip arthroplasty. Similar findings were reported by Kwak et al., who noted that conventional fixation techniques might provide less stability in complex acetabular fractures involving the quadrilateral surface, and emphasized the need for modifications to improve support [7].

In recent years, new implant designs have focused on improving anatomical fit and biomechanical stability. Studies by Wang et al. [8] Yalın Kılınç et al. [9] have shown that the use of anatomical plates designed specifically for the quadrilateral surface, such as the square bracket-shaped tubular plate, can provide better support while reducing the risk of complications like joint penetration. These implants, often used in combination with the modified Stoppa or ilioinguinal approach, have demonstrated promising results, reducing the need for revision surgeries and improving functional outcomes.

However, despite these advancements, there are still challenges with plate fit, particularly when anatomical variability in the quadrilateral surface leads to a mismatch between the implant and bone structure. Studies by Wang and colleagues and Nadeem et al. emphasized the importance of customized implants to ensure proper fixation and to minimize the risk of complications [8,10,11].

Elderly patients present unique challenges in the management of acetabular fractures [12]. In particular, the risk of post-traumatic arthritis and the potential need for subsequent hip replacement procedures is higher in this demographic. Our own cohort included a patient who required total hip arthroplasty due to complications arising from inadequate fixation [13]. This is consistent with the findings of Peter, who observed that while good clinical outcomes can be achieved with the ilioinguinal approach and quadrilateral surface plates, a significant proportion of elderly patients may eventually require total hip arthroplasty due to the long-term effects of poorly stabilized fractures [14].

Biomechanical studies, such as those conducted by Kwak et al., have provided valuable insights into the stability of various fixation methods. Their findings indicate that while traditional suprapectineal pelvic brim plates provide greater stiffness and stability, newer anatomical QLS plates, though effective, may result in increased medial displacement of posterior column fragments [15]. This highlights the ongoing need for further innovations in plate design to optimize fixation strength and anatomical alignment.

Our study presents encouraging outcomes. However, we recognize the limitation of the small number of patients and the limited follow-up period. 

## Conclusions

The fixation of quadrilateral surface fractures remains a critical aspect of acetabular fracture management, particularly with the increasing number of cases in elderly patients. Our study, along with the findings from other studies, emphasizes the importance of adequate medial buttressing and the use of well-designed, cost-effective fixation techniques. Future research should focus on improving implant design and optimizing surgical techniques to reduce complications and improve long-term outcomes for patients with acetabular fractures involving the quadrilateral surface.

## References

[1] Tosounidis TH, Giannoudis PV (2015). What is new in acetabular fracture fixation?. Injury.

[2] Ochs BG, Marintschev I, Hoyer H, Rolauffs B, Culemann U, Pohlemann T, Stuby FM (2010). Changes in the treatment of acetabular fractures over 15 years: analysis of 1266 cases treated by the German Pelvic Multicentre Study Group (DAO/DGU). Injury.

[3] Chen K, Yao S, Yin Y (2023). A new classification for quadrilateral plate fracture of acetabulum. Injury.

[4] Cole JD, Bolhofner BR (1994). Acetabular fracture fixation via a modified Stoppa limited intrapelvic approach. Description of operative technique and preliminary treatment results. Clin Orthop Relat Res.

[5] Schäffler A, Freude T, Stuby F, Höntzsch D, Veltkamp J, Stöckle U, König B (2016). Surgical treatment of acetabulum fractures with a new acetabulum butterfly plate (in German). Z Orthop Unfall.

[6] Ciolli G, De Mauro D, Rovere G (2021). Anterior intrapelvic approach and suprapectineal quadrilateral surface plate for acetabular fractures with anterior involvement: a retrospective study of 34 patients. BMC Musculoskelet Disord.

[7] Kwak DK, Lee SH, Lee KU, Hwang JH, Yoo JH (2022). Simultaneous reduction and fixation using an anatomical suprapectineal quadrilateral surface plate through modified Stoppa approach in superomedially displaced acetabular fractures. Sci Rep.

[8] Wang W, Cai X, Liu X, Wang G, Kang H, Qian S (2024). Special contoured pelvic brim reconstruction titanium plate combined with trans-plate buttress screws (quadrilateral screws) for acetabular fractures with quadrilateral plate involvement through the anterior ilioinguinal approach. Front Surg.

[9] Kilinç CY, Gültaç E, Can Fİ, Kilinç RM, Şener B, Açan AE (2024). Comparison of 2 different fixation techniques of comminuted acetabular quadrilateral surface fractures using square bracket-shaped tubular plate or interfragmentary screws in addition to supra/infrapectineal plate fixation: an observational study. Medicine (Baltimore).

[10] Nadeem U, Qadir I, Mazari J, Zaman AU, Aziz A (2021). Outcomes of direct infrapectineal buttress plate for quadrilateral surface fractures of acetabulum using an anterior intrapelvic approach. Hip Pelvis.

[11] Procaccini R, Pascarella R, Carola D (2022). The use of suprapectineal plate in acetabular fractures via ilioinguinal approach with Stoppa window. Orthop Rev (Pavia).

[12] Karim MA, Abdelazeem AH, Youness M, El Nahal WA (2017). Fixation of quadrilateral plate fractures of the acetabulum using the buttress screw: a novel technique. Injury.

[13] Laflamme GY, Hebert-Davies J, Rouleau D, Benoit B, Leduc S (2011). Internal fixation of osteopenic acetabular fractures involving the quadrilateral plate. Injury.

[14] Peter RE (2015). Open reduction and internal fixation of osteoporotic acetabular fractures through the ilio-inguinal approach: use of buttress plates to control medial displacement of the quadrilateral surface. Injury.

[15] Kwak DK, Jang JE, Kim WH, Lee SJ, Lee Y, Yoo JH (2023). Is an anatomical suprapectineal quadrilateral surface plate superior to previous fixation methods for anterior column-posterior hemitransverse acetabular fractures typical in the elderly?: A biomechanical study. Clin Orthop Surg.

